# Prevention of thromboembolism in spinal cord injury -S1 guideline

**DOI:** 10.1186/s42466-020-00089-7

**Published:** 2020-12-10

**Authors:** Norbert Weidner, Oliver J. Müller, Viola Hach-Wunderle, Karsten Schwerdtfeger, Rüdiger Krauspe, Rolf Pauschert, Christian Waydhas, Michael Baumberger, Christoph Göggelmann, Gabriela Wittgruber, Renate Wildburger, Oswald Marcus

**Affiliations:** 1 Klinik für Paraplegiologie, Universitätsklinikum Heidelberg, Schlierbacher Landstrasse 200a, 69118 Heidelberg, Germany; 2 Klinik für Innere Medizin III, Universitätsklinikum Schleswig-Holstein, Campus Kiel, Kiel, Germany; 3 Klinik für Gefäßchirurgie und Gefäßmedizin, Krankenhaus Nordwest, Frankfurt/M., Germany; 4 Klinik für Neurochirurgie, Universitätsklinikum des Saarlandes, Homburg Saar, Germany; 5 Klinik und Poliklinik für Orthopädie, Universitätsklinikum, Heinrich-Heine-Universität Düsseldorf, Düsseldorf, Germany; 6 Fachabteilung für Orthopädie/Unfallchirurgie, SRH Gesundheitszentrum Bad Wimpfen, Bad Wimpfen, Germany; 7 Berufsgenossenschaftliches Universitätsklinikum Bergmannsheil, Bochum, Germany; 8 Paraplegikerzentrum, Nottwil, Schweiz, Nottwil, Switzerland; 9 Klinik für Innere Medizin III: Kardiologie, Angiologie und Pneumologie, Universitätsklinikum Heidelberg, Heidelberg, Germany; 10 Rehabilitationsklinik Tobelbad, Tobelbad, Austria; 11 BG Unfallklinik Frankfurt/M., Frankfurt/M., Germany

## Abstract

**Introduction:**

Traumatic and non-traumatic spinal cord injury bears a high risk for thromboembolism in the first few months after injury. So far, there is no consented guideline regarding diagnostic and prophylactic measures to prevent thromboembolic events in spinal cord injury. Based on a Pubmed research of related original papers and review articles, international guidelines and a survey conducted in German-speaking spinal cord injury centers about best practice prophylactic procedures at each site, a consensus process was initiated, which included spinal cord medicine experts and representatives from medical societies involved in the comprehensive care of spinal cord injury patients. The recommendations comply with the German S3 practice guidelines on prevention of venous thromboembolism.

**Recommendations:**

Specific clinical or instrument-based screening methods are not recommended in asymptomatic SCI patients. Based on the severity of neurological dysfunction (motor completeness, ambulatory function) low dose low molecular weight heparins are recommended to be administered up to 24 weeks after injury. Besides, mechanical methods (compression stockings, intermittent pneumatic compression) can be applied. In chronic SCI patients admitted to the hospital, thromboembolism prophylactic measures need to be based on the reason for admission and the necessity for immobilization.

**Conclusions:**

Recommendations for thromboembolism diagnostic and prophylactic measures follow best practice in most spinal cord injury centers. More research evidence needs to be generated to administer more individually tailored risk-adapted prophylactic strategies in the future, which may help to further prevent thromboembolic events without causing major side effects. The present article is a translation of the guideline recently published online (https://www.awmf.org/uploads/tx_szleitlinien/179-015l_S1_Thromboembolieprophylaxe-bei-Querschnittlaehmung_2020-09.pdf).

## Introduction

The present article is a translation of the guideline recently published online (https://www.awmf.org/uploads/tx_szleitlinien/179-015l_S1_Thromboembolieprophylaxe-bei-Querschnittlaehmung_2020-09.pdf). The annual incidence of deep leg vein thrombosis (DVT) varies between 90 and 130 per 100,000 persons, equivalent to an average of 0.1% in the general population [13, 22]. The frequency of symptomatic and asymptomatic DVT in surgical and non-surgical medicine without prophylaxis (prevalence) arises as follows [13]: Internal medicine diseases 10–20%, stroke 20–50%, polytrauma 40–80%, and spinal cord injury 60–80%. Venous thromboembolism (VTE) is a common complication in spinal cord injury with resulting paresis. Of all traumatic injuries, traumatic spine injuries affecting the spinal cord and/or the cauda equina bear the highest risk for VTE [9]. Reasons for this are the failure of the muscle pump due to the paresis, a presumed transient hypercoagulative phase, and accompanying endothelial damage. Decoupling from supraspinal control is also discussed as a relevant prothrombotic factor [21]. Pulmonary embolism, leg edema caused by postthrombotic syndrome and hemorrhage due to the required anticoagulation, represent complications of VTE. Also, patients can be burdened by frequently required laboratory controls undergoing therapy with vitamin K antagonists.

The frequency of VTE (particularly in the English literature the terms frequency, incidence, risk, and rate are used interchangeably) varies extremely depending on the study between 49 and 100% (older studies) or 1.6 to 45% (recent studies published between 2010 and 2019) in acute SCI (within 3 months after the date of injury), depending on the detection method, the observation period and the implementation of prophylactic measures [22]. The risk (calculated in 3-month hazard intervals) for a VTE decreases with time since injury/disease onset [11]. Thus, the risk of VTE is 34% within the first 3 months, 1.1% after 6 months, and 0.4% after 1 year. The neurological level of injury affects the likelihood of VTE. In paraplegic patients with a high thoracic injury level, higher incidences were found compared to quadriplegics: In quadriplegics with a level between C1 and C4, the incidence is 3.4%. Paraplegics with level T1-T6, on the other hand, have an almost twice as high incidence of 6.3%. Low thoracic (T7–12) or lumbar injury levels are associated with incidences comparable to cervical injuries (4.47 and 3.17%, respectively). The reason for the VTE cluster in high thoracic SCI has yet to be determined [20]. The more pronounced the neurological deficits according to the American Spinal Injury Association Impairment Scale (AIS) are, the higher is the risk of suffering from VTE. The risk is particularly high in sensorimotor complete SCI. No evidence is available regarding the incidence of VTE in flaccid paralysis (lower motor neuron lesion due to conus medullaris and/or cauda equina lesion – typically caudal to the Th12 vertebral body) versus spastic paralysis (upper motor neuron lesion due to a spinal cord lesion – typically C1 and Th10–12 vertebral bodies). However, the absence of a spastic tone increase has been described as a risk factor for VTE [6]. Concerning age, a study of more than 12,000 patients showed no correlation with VTE events [11]. A previous VTE increases the risk of recurrent VTE by a factor of 6 [10]. If thrombosis prophylaxis was started within the first 2 weeks after the onset of paraplegia, a significantly reduced risk of VTE was shown compared to a delayed start of prophylaxis [25]. In respect to pre-existing thrombophilia, a possibly increased risk is described in the presence of a prothrombin or factor V Leiden gene mutation, antithrombin, protein C/protein S deficiency, hyperhomocysteinemia, or persistently increased factor VIII levels. However, so far neither the independent predictive value of a hereditary thrombophilia nor the clinical significance of the factors mentioned could be confirmed [3]. The factors gender, obesity, alcohol/nicotine consumption, and insurance status do not show a clear correlation with the risk of VTE [11], even if this is described in a retrospective analysis with a relatively low number of cases [24]. In summary, paraplegia per se carries a high risk of VTE, regardless of the cause or concomitant disease.

The present guideline provides a concise overview of the risk of VTE and recommendations regarding diagnosis and prophylactic measures to prevent VTE in acute and chronic SCI. The pattern of paralysis is defined by the severity and level of SCI. The different types of paralysis require to properly adjust the method and duration of prophylaxis. Additional specific risk factors and their relevance for VTE prophylaxis in spinal cord injury need to be considered. The scope of the guideline extends to traumatic and non-traumatic SCI. The guideline covers the time from the subacute (patient transfer to dedicated spinal cord injury center typically within the first 1–4 weeks after injury) to the chronic phase (readmission for treatment of secondary complications of SCI). The acute phase immediately after injury will be addressed in a separate guideline.

## Methods of guideline development

The S1 level guideline (AWMF registry No. 179–015), valid until August 30, 2025, is based on a systematic Pubmed search of original papers and review articles including the keywords spinal cord injury, prophylaxis, thrombosis, thromboembolism. Moreover, the extensive review by members of the Spinal Cord Injury Research Evidence (SCIRE) initiative on the topic “venous thromboembolism following spinal cord injury” was taken into account [22]. SCIRE reviews and rates scientific research on SCI rehabilitation to make quality information more available (https://scireproject.com). The present guideline has been harmonized with the already existing S3 AWMF guideline “prophylaxis of venous thromboembolism” [13] and the US American guideline of the Consortium for Spinal Cord Medicine “Prevention of thromboembolism in spinal cord injury” [3]. Finally, a web-based survey by the DMGP was carried out among a total of 26 German-speaking spinal cord injury centers (February 5–25, 2017), the results of which were also taken into account in this guideline.

A group of experts in spinal cord medicine together with delegates from relevant medical societies (Deutschsprachige Medizinische Fachgesellschaft für Paraplegiologie, Deutsche Gesellschaft für Angiologie, Deutsche Gesellschaft für Innere Medizin, Deutsche Gesellschaft für Neurochirurgie, Deutsche Gesellschaft für Neurologie, Deutsche Gesellschaft für Orthopädie und Unfallchirurgie) developed a guideline, which was consented by all participating medical societies.

## Recommendations

### Diagnosis - background

In principle, the rationale for screening tests to detect asymptomatic VTE is valid, in particular in patients with SCI. However, studies have shown that neither regular clinical examination of the lower extremities nor D-dimer analysis or ultrasound scans have sufficient sensitivity and specificity to justify screening. In a Japanese study, elevated D-dimer levels (> 16 μg/dl) 2 weeks after trauma were postulated as a good predictor of VTE [19]. However, sensitivity and specificity were relatively low with 77.3 and 69.2%, respectively, and it was a laboratory analysis within a very tight timeframe that is not applicable to most clinical situations. None of the patients with VTE had received anticoagulant VTE prophylaxis, which is not in line with common practice in German-speaking hospitals. It is still unclear how the suggested D-dimer level compares to currently used highly sensitive D-dimer tests. In another Japanese study, increased D-dimer levels (> 10 μg/ml) were found in 20% of patients in a rehabilitation clinic. In two-thirds of these patients, an asymptomatic VTE was found in the ultrasound examination [17]. In a subacute rehabilitation setting, a retrospective examination showed that a D-dimer screening at admission (performed on average almost 7 weeks after the date of injury) indicated clinically inapparent thromboembolism in 5 of 8 cases, which was then confirmed by ultrasound, phlebography, or CT [7]. However, the clinical relevance of asymptomatic ultrasound findings is still unclear. Routine screening by ultrasound shows a low sensitivity between 29 and 31%, thus a high rate of false-positive findings compared to intravenous phlebography [29, 30]. The side effects of anticoagulant thrombosis prophylactic measures, especially bleeding events, must be taken into account [7]. Furthermore, clinically apparent/symptomatic events of a VTE are not prevented despite screening [26]. For these reasons, regular screening by ultrasound and D-dimer testing is not recommended in the Consortium for Spinal Cord Medicine guideline [3].

### Diagnosis – recommendation

A general clinical, laboratory (D-dimer testing) or instrument-based screening (e.g. ultrasound) for DVT/VTE is not recommended in asymptomatic SCI patients.

### Prophylactic therapy in subacute spinal cord injury - background

The S3 guideline for VTE prophylaxis states: “No reliable tests to determine the individual risk of thrombosis are available so far. However, the fact that asymptomatic thrombosis can cause a postthrombotic syndrome and that the vast majority of fatal pulmonary embolisms occur without clinical announcement, justify general thromboembolic prophylaxis in defined risk situations” [13]. With the date of SCI and resulting paresis a clear “risk situation” has occurred and thus prophylactic measures are indicated.

In principle, the following measures are available for VTE prophylaxis: Basic, mechanical, and anticoagulant methods [13].

### Basic methods

General basic measures include, in addition to the earliest possible mobilization carried out as part of the acute treatment, the best possible activation of the patients through manually assisted and machine-supported movement exercises as well as instructions for self-exercise and sufficient hydration.

### Anticoagulant methods

In principle, heparins (unfractionated, low-molecular-weight heparins), danaparoid, factor Xa inhibitors, thrombin inhibitors, and vitamin K antagonists are approved for VTE prophylaxis [13]. In the technical information for each low-molecular-weight heparin (LMWH), different indications and contraindications are listed, some of which specifically rule out VTE prophylaxis with LMWH (e.g. dalteparin, nadroparin) in SCI (contraindication: injuries and surgical interventions in the central nervous system). Of note, paraparesis due to injury to structures of the peripheral nervous system such as cauda equina syndrome is not listed as a contraindication. However, the listed indication “Prophylaxis of venous thromboembolic disease in patients with acute disease and limited mobility with increased risk of venous thromboembolism” covers VTE prophylaxis with LMWH in SCI.

According to a systematic review, low dose unfractionated heparin (UFH) alone cannot reduce the DVT rate in SCI [22]. Only in combination with electrostimulation, low dose UFH showed a significant thrombosis prophylactic effect. After the administration of an adjusted dose of UFH, a thrombosis prophylactic effect could be demonstrated, but there was an increased incidence of bleeding complications [22]. There is no study on LMWH which has investigated LMWH administration versus no LMWH administration without mechanical methods in SCI. In a cohort of acute SCI, the administration of low dose LMWH (enoxaparin 40 mg 1x/die) in combination with mechanical methods (compression treatment - not exactly defined) reduced the DVT rate (predominantly asymptomatic) from 22 to 5% compared to compression treatment alone [14].

In general, there is no clear evidence from a prospective study demonstrating the superiority of low dose LMWH over low dose UFH [18]. However, smaller prospective and more comprehensive retrospective studies show a trend towards higher thromboprophylactic efficacy of low dose LMWH [22]. Bleeding complications tend to occur more frequently under UFH [22].

Retrospective studies have not shown that an increased dose of LMWH is more effective than the prophylactic standard dose [15]. There is also no evidence to date regarding the efficacy of different LMWH [22]. Danaparoid, a heparin-free mixture of heparinoids, shows a good VTE prophylactic effect (study population without SCI) and can be used in particular for heparin-induced thrombopenia type II (HIT II) [13]. Fondaparinux has proven its VTE prophylactic effect in clinical studies (study population without SCI) and is an alternative to heparins; the production by genetic engineering minimizes the risk for a HIT [13]. According to the DMGP survey 2017, anticoagulant VTE prophylaxis with low-molecular-weight heparin in prophylactic standard dosage is carried out in 92% of the surveyed centers in acute motor complete and incomplete SCI.

Deviating doses of LMWH apply to certain risk groups; analysis of the anti-factor Xa peak level may then be helpful. In the case of obesity (BMI > 35 kg/m^2^ or bodyweight > 95 kg) a dose increase is justified, but there is no sufficiently validated dosage recommendation. In patients with low bodyweight, an adjustment of the LMWH dose is recommended. In case of renal failure with a GFR < 30 ml/min, the indication should be reevaluated or the dose of LMWH should be reduced (according to the manufacturer’s recommendation). In case of a GFR < 15–20 ml/min, LMWH administration is only recommended on a case by case basis or a prophylactic dose of UFH can be administered alternatively.

Non-vitamin K dependent oral factor Xa inhibitors (direct oral anticoagulants - DOAC; apixaban, rivaroxaban, edoxaban) and the thrombin inhibitor dabigatran etexilate provide effective VTE prophylaxis after hip and knee arthroplasty [1]. To date, there are no studies on the effectiveness in SCI. Thus far, edoxaban has received medical approval only in Japan [13].

There are no studies available regarding the VTE prophylactic effects of vitamin K antagonists in acute SCI. However, these substances are considered rather unfavorable because of the increased risk of bleeding, frequent laboratory controls as well as their prolonged effects [3]. Moreover, Vitamin K antagonists are not approved for VTE prophylaxis.

In case of serious bleeding complications under anticoagulants, antagonization with protamine (heparins), with vitamin K or PPSB (vitamin K antagonists) or with specific antidotes (andexanet-alfa for factor Xa inhibitors and idarucizumab for dabigatran) or unspecific measures (PPSB for all factor Xa inhibitors/thrombin inhibitor) can be administered [13]. Regarding the procedure for HIT, we refer to the current S3 guideline [13].

In SCI, especially as a result of spine trauma or underlying intraspinal hemorrhage, an increased risk of bleeding must be taken into account in VTE prophylaxis with anticoagulants. If necessary, mechanical methods should be used preferably or exclusively if there is acute bleeding or an acute risk of bleeding [13]. Besides, muscular hematomas in the thigh area are sometimes observed as a complication of para- or tetraplegia in the course of acute medical or rehabilitative treatment (expert opinion). In this case, a temporary discontinuation of VTE prophylaxis with anticoagulants should be considered and mechanical methods should be applied alternatively.

### Mechanical methods - intermittent pneumatic compression (IPC)

IPC replaces the work of the calf muscle pump in immobilized or paralyzed patients. For VTE prophylaxis, devices with 1–3 chambers are used. After applying the foot or leg cuffs, the air chambers are automatically inflated and deflated [13]. In SCI, only a small case series has been described in which patients received IPK in combination with compression stockings without prophylactic anticoagulation [4]. Here, a high (mostly asymptomatic) VTE rate of 43% was observed. Evidence of effectiveness is only available from non-traumatic patient cohorts and following joint replacement surgery (without paresis) [5, 16]. According to this, IPC can be considered as effective as a pharmacological prophylaxis, however, with a lower risk of bleeding [13]. In a systematic review, no clear advantages of a combination of IPC with anticoagulant VTE prophylaxis could be demonstrated compared to VTE prophylaxis with LMWH alone [2]. According to the 2017 DMGP survey, no center currently uses intermittent pneumatic compression. The low acceptance of IPC is likely due to the fact that rehabilitative interventions, which are implemented early after injury, are limited while IPC is applied.

### Mechanical methods - compression stockings

A distinction is made between graduated compression stockings (GCS) and medical thrombosis prophylaxis stockings (MTPS). Neither GCS nor MTPS have an evidence base concerning their VTE prevention efficacy in SCI patients. In principle, an effective risk reduction with regard to DVT was found in a non-SCI study cohort (meta-analysis) [27]. However, the effects were moderate. At the same time, the risk of skin irritation (high risk in SCI) and the contraindication for significant peripheral arterial occlusive disease must be considered. The combination of MTPS with LMWH (see above) versus compression with MTPS alone results in significantly lower DVT rates in SCI [14]. No reliable evidence is available for thigh-length versus calf-length MTPS regarding their thromboprophylactic effectiveness [28]. In cases of marked edema in the paretic lower extremities, thigh-length MTPS may be preferred, although they can also lead to constrictions and skin abrasions.

According to the 2017 DMGP survey, compression stockings are implemented for acute motor complete SCI in 91% and for incomplete SCI in 88% of the centers that participated in the survey (58% GCS, 21% one layer of MTPS, and 13% two layers of MTPS). In particular, when two layers of MTPS are used, frequent skin checks (at least twice a day) must be carried out to detect or prevent skin damage at an early stage, especially at the predilection sites (popliteal fossa, back of the foot, heel) (expert opinion).

### Mechanical methods - AV impulse system

Intermittent foot compression increases the venous blood flow in proximal veins. Following unilateral hip surgery, no advantage of an AV impulse system in combination with LMWH was found compared to LMWH alone [27]. To date, there are no studies available for SCI. According to the DMGP survey 2017, AV impulse systems are not used at all.

In summary, although mechanical methods are an effective basic VTE prophylaxis, the risk of VTE is increased when mechanical methods are administered alone compared to anticoagulant prophylaxis with heparins [8]. Thus, in the absence of contraindications, anticoagulant prophylaxis should be preferred. However, if there is a high risk of bleeding and anticoagulant prophylaxis cannot be used, mechanical methods are indicated.

### Vena cava filter

There is no evidence to justify the application of a vena cava filter in SCI [3]. Conversely, there is evidence that the prophylactic implantation of vena cava filters during rehabilitation treatment is associated with an even higher risk of VTE [12]. Relevant subgroups that could benefit from filter implantation have not been identified to date.

### Duration of thrombosis prophylaxis

There are no definitive studies on the duration of VTE prophylaxis. In principle, anticoagulant VTE prophylaxis should be based on the persistence of relevant risk factors for VTE [13]. When assessing the duration of VTE prophylaxis in SCI, the high risk of VTE within the first 3 months should be considered [10, 11].

Sixty-two percent of the surveyed German-speaking spinal cord injury centers use anticoagulant VTE prophylaxis for motor complete SCI for a period of 12 weeks, 27% for longer than 12 weeks and 7% for only up to 6 weeks. In the case of motor incomplete SCI, prophylaxis is carried out for a total of 12 weeks in 69% of the centers, and in 12% longer than 12 weeks and up to 6 weeks, respectively.

Thirty-eight percent of the centers apply mechanical methods in addition to anticoagulant prophylaxis in motor complete SCI for 12 weeks and 42% for longer than 12 weeks. In the case of motor incomplete SCI, mechanical methods are applied for a total of 12 weeks in 58% of the centers and in 19% for longer than 12 weeks.

#### Prophylactic therapy in subacute spinal cord injury - recommendations

**Fig. 1:**
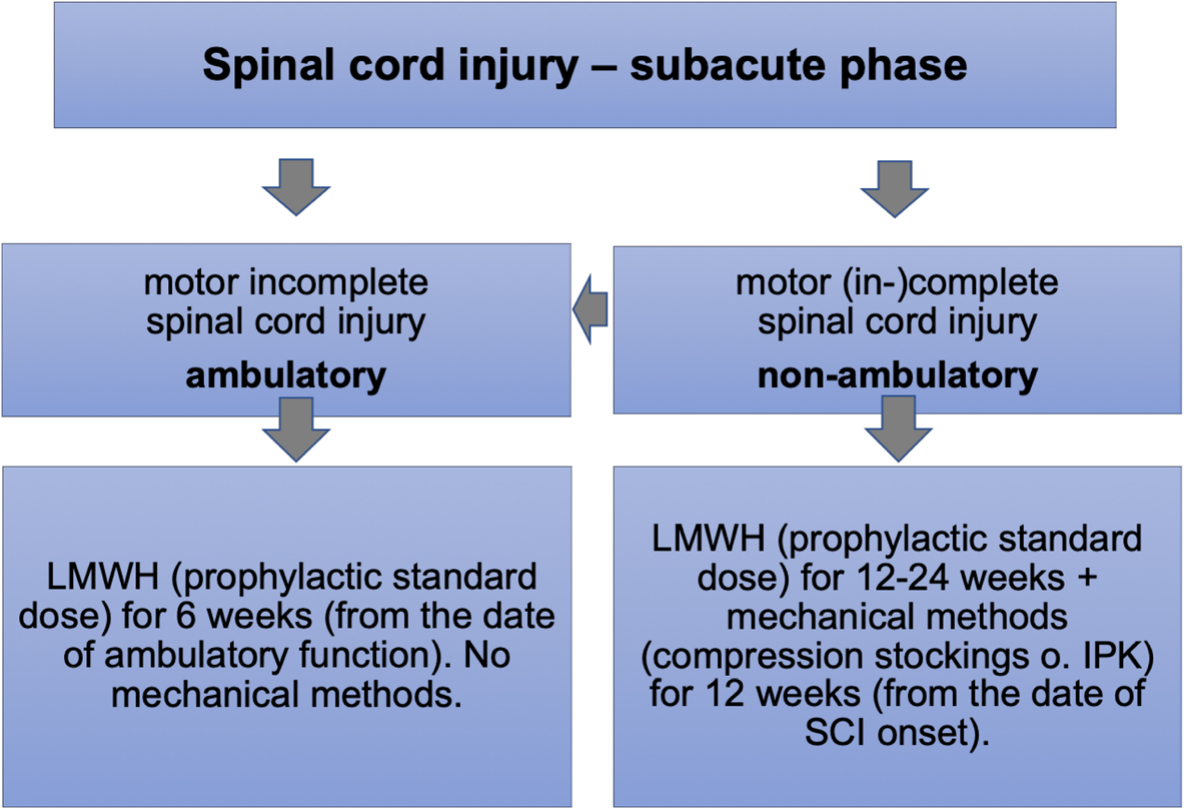
Flow chart VTE prophylaxis in subacute SCI

- ▪ VTE prophylactic measures for SCI should be applied in analogy to diseases with a high risk of VTE [13], but taking into account the individual etiology. For example, in SCI caused by spinal hemorrhage with consecutive compression of the spinal cord, anticoagulant VTE prophylaxis should be temporarily reduced or suspended.
- ▪ In cases of motor (in-)complete SCI, LMWH should be administered in standard prophylactic doses (expert information for the respective LMWH needs to be considered) over 12–24 weeks from the onset of SCI. In addition, mechanical methods (compression stockings or IPC) can be applied over 12 weeks from the onset of SCI under consideration of contraindications. Particular awareness should be given to the increased risk of pressure sores caused by mechanical methods.
- ▪ In case of motor incomplete SCI with preserved/regained walking function (at least able to walk with a walker, regardless of walking distance, walking time, or therapist support), LMWH should be administered in a standard prophylactic dosage for at least 6 weeks (calculated from the time of regained ability to walk), but no longer than 24 weeks after the onset of SCI. Additional mechanical methods can be applied if the ability to walk has not yet been regained, but no longer than 12 weeks after the onset of SCI.
- ▪ Mechanical methods (compression stockings or IPC) should be applied if there are contraindications against anticoagulant VTE prophylaxis.
- ▪ No general recommendation can be made for vitamin K antagonists or DOAC (factor Xa inhibitors, factor II inhibitor). However, these can be considered as alternatives to LMWH after completion of relevant acute medical interventions (especially surgery). In the case of off-label use of an oral Factor Xa inhibitor for primary prophylaxis, a reduced dose of apixaban (2 × 2.5 mg) or rivaroxaban (1x10mg)) approved for long-term secondary prophylaxis may be used (Fig. 1).

#### Prophylactic therapy in chronic spinal cord injury - background

It can be assumed that individuals with chronic SCI have a risk of VTE at inpatient readmission comparable to that of patients without SCI. A retrospective examination of patients with chronic SCI found an incidence of VTE at hospitalization due to plastic-reconstructive surgery of 0.2% compared to 1.7% in a general surgical control cohort [23].

In hospitalized chronic SCI patients, 80% of the surveyed German-speaking spinal cord injury centers generally apply anticoagulant VTE prophylaxis with LMWH in chronic motor complete SCI and 77% in motor incomplete SCI respectively. Sixty-two percent of the centers initiate anticoagulant VTE prophylaxis dependent on the circumstances for admission (e.g. surgical intervention, immobilization, acute illness). Fifty percent of the centers generally use GCS, 54% do so depending on the reason for admission.

#### Prophylactic therapy in chronic spinal cord injury - recommendation

- ▪ Ambulatory and non-ambulatory in-patients with SCI, immobilized: LMWH in standard prophylactic dosage, taking into account any contraindications.
- ▪ Ambulatory and non-ambulatory in-patients with SCI, mobilized: Thrombosis prophylaxis according to S3 guideline VTE prophylaxis [13].

## Data Availability

Not applicable.
